# Green Topochemical Esterification Effects on the Supramolecular Structure of Chitin Nanocrystals: Implications for Highly Stable Pickering Emulsions

**DOI:** 10.1021/acsanm.1c03708

**Published:** 2022-04-04

**Authors:** Chiara Magnani, Mina Fazilati, Roland Kádár, Alexander Idström, Lars Evenäs, Jean-Marie Raquez, Giada Lo Re

**Affiliations:** † Laboratory of Polymeric and Composite Materials (LPCM), Center of Innovation and Research in Materials & Polymers (CIRMAP), University of Mons (UMONS), B-7000 Mons, Belgium; ‡ Laboratory of Proteomics and Microbiology, Research Institute for Biosciences, University of Mons (UMONS), B-7000 Mons, Belgium; § Department of Industrial and Materials Science IMS, Chalmers University of Technology, SE-412 96 Gothenburg, Sweden; ∥ Wallenberg Wood Science Center (WWSC), Chalmers University of Technology, SE-412 96 Gothenburg, Sweden; ⊥ Department of Chemistry and Chemical Engineering, Chalmers University of Technology, SE-412 96 Gothenburg, Sweden

**Keywords:** supramolecular organization, chitin nanocrystals, topochemistry, nematic structures, colloidal rheology, Pickering emulsions, solid-state NMR

## Abstract

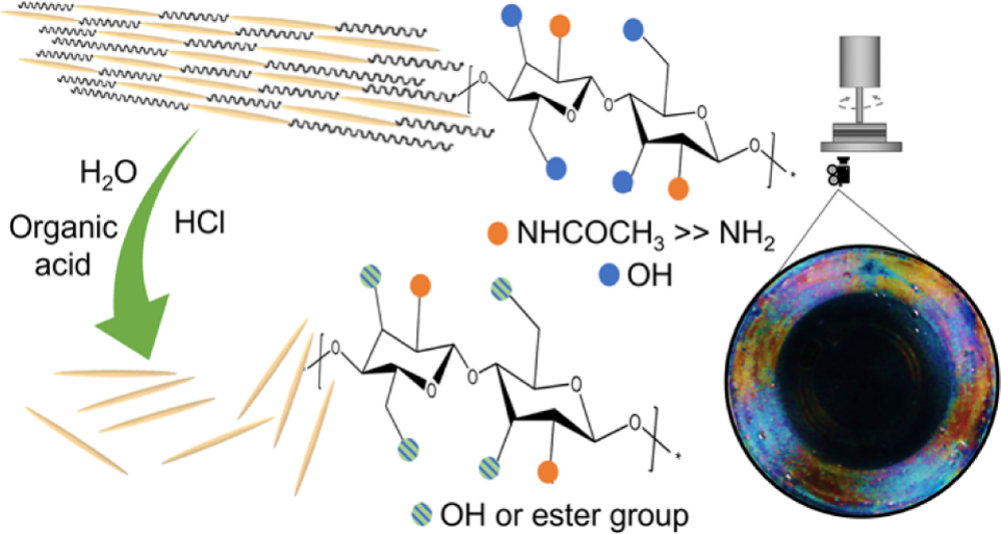

In nature, chitin is organized in hierarchical structures composed of nanoscale building blocks that show outstanding mechanical and optical properties attractive for nanomaterial design. For applications that benefit from a maximized interface such as nanocomposites and Pickering emulsions, individualized chitin nanocrystals (ChNCs) are of interest. However, when extracted in water suspension, their individualization is affected by ChNC self-assembly, requiring a large amount of water (above 90%) for ChNC transport and stock, which limits their widespread use. To master their individualization upon drying and after regeneration, we herein report a waterborne topochemical one-pot acid hydrolysis/Fischer esterification to extract ChNCs from chitin and simultaneously decorate their surface with lactate or butyrate moieties. Controlled reaction conditions were designed to obtain nanocrystals of a comparable aspect ratio of about 30 and a degree of modification of about 30% of the ChNC surface, under the rationale to assess the only effect of the topochemistry on ChNC supramolecular organization. The rheological analysis coupled with polarized light imaging shows how the nematic structuring is hindered by both surface ester moieties. The increased viscosity and elasticity of the modified ChNC colloids indicate a gel-like phase, where typical ChNC clusters of liquid crystalline phases are disrupted. Pickering emulsions have been prepared from lyophilized nanocrystals as a proof of concept. Our results demonstrate that only the emulsions stabilized by the modified ChNCs have excellent stability over time, highlighting that their individualization can be regenerated from the dry state.

In nature, nanoscale building blocks self-assemble to create hierarchically structured biomaterials.^1^ The underlying supramolecular interactions provide outstanding mechanical and optical properties to cellulose and chitin, making plants and arthropods an endless source of inspiration for nanomaterial design.^2^ Chitin represents the second most available biopolymer, and, like cellulose in wood, it is the main structural component in mushrooms^3^ and exoskeletons of arthropods.^4^ Semicrystalline chitin nanofibrils are held together by hydrogen bonds, which are supramolecularly organized in micro-sized bundles embedded with proteins.^4^ Thanks to this organization, the exocuticle of crustaceans can reach a stiffness of 8.5–9.5 GPa.^4^ Due to the abundance and renewability of chitin, various methods to isolate its nanocrystals have been developed, using water as main extractive medium.^5,6^ Nanosized inherent properties such as high surface areas and reactivity, together with the mechanical performance and antibacterial activity,^7,8^ have led to the exploitation of nano-chitin in pharmaceutical,^9^ biomedical,^10,11^ cosmetic,^12^ and food-related^13^ applications, among others.^14^

Chitin nanocrystal (ChNC) potential can be exploited in different application categories. On one hand, the anisotropic morphology of ChNCs through their self-assemblies generates liquid crystal structures,^15,16^ being interesting for optic, photonic, and biomimetic fields.^2,17^ On the other hand, individualized ChNCs are desired where the interactions with the surrounding medium should be maximized. For example, in nanocomposites and Pickering emulsions, individualization boosts nucleation,^18^ reinforcing,^19,20^ or stabilizing effects.^21−24^

The exploitation of ChNCs in applications where their individualization is crucial demands the mastering of their self-assembly, governed by their interactions in water suspension^15^ or upon drying.^19,21^ It is worth to highlight that the amount of water required to avoid self-assembly prior the ChNC use is above 90%, representing the main limits of the ChNC widespread use. At the same time, water removal leads to irreversible self-assembly, hindering their regeneration from the dried state as individualized nanocrystals.

Properties on the nanoscale are governed by the surface, which finely tunes ChNC mutual interactions with the surrounding media.^25^ “God made the bulk; surfaces were invented by the devil” Wolfgang Pauli commented, predicting the inherent challenges in mimicking hierarchical structure assemblies.^2^ Furthermore, to discern between individualized or self-assembled structures is still far to be trivial, and adapted methodologies are required.

Nature suggests stunts to regulate the self-organization of hierarchical systems. Topochemical acetylation of polysaccharides is used in plants to tune cellulose functions in cell walls and regulate the sensitivity to water and hence their ultimate degradation rate.^26,27^ In arthropods, chitin is partially deacetylated^28^ to tune the interactions with different proteins,^29^ which regulate the periodic distance in the supramolecular organization of chitin.^2^ This distance, commonly called “pitch”, determines the hardness versus softness of the tissue formed by the layered chitin sheets, differentiating its specific function.^4^

Here, we have explored the relevance of the topochemical modification in the ChNC nematic structuring and its colloidal rheological behavior. Our study aims to elucidate the mechanism of ChNC self-assemblies on the nanoscale that would allow the control of nanocrystal individualization.

Although some literature studies have evaluated ChNC surface modification for different purposes,^14^ like improving compatibility in nanocomposites,^30−32^ only few papers have assessed its effect on self-assembly in colloidal systems. Tzoumaki *et al.*,^33^ and later others,^15,16,19^ have studied the effect of different parameters, like concentration, pH, ionic strength, and temperature, on nano-chitin self-assemblies. Their impact on the pitch of the chiral nematic phase has been described by Narkevicius *et al.*(15) However, to our knowledge, after the first observation of Li *et al.* on the effect of the acetylation degree,^34^ the effect of ChNC topochemistry toward their self-organization has not been reported.

Grounded on a more general hypothesis that ChNC topochemical modification can lead to effectively master their self-assembly in water suspension and upon drying, we propose here a new green one-pot acid hydrolysis/Fischer esterification method for the surface modification of ChNCs, adapted from previously prepared cellulose nanocrystals (CNCs).^35−37^

This method allows the extraction of nanocrystals and the simultaneous esterification of their surface hydroxyl groups with a renewable organic acid *via* a catalytic amount of hydrochloric acid (HCl). Under the rationale that tuning the hydroxyl functionalities would impact the nanocrystal self-assembly,^21,38^ butyric and lactic acids have been selected for esterification. Although the introduced hydrophobic moieties add comparable steric hindrance, only lactate reintroduces a hydroxyl group, which is conceivably available for further hydrogen bond formation (Figure 1a).

**Figure 1:**
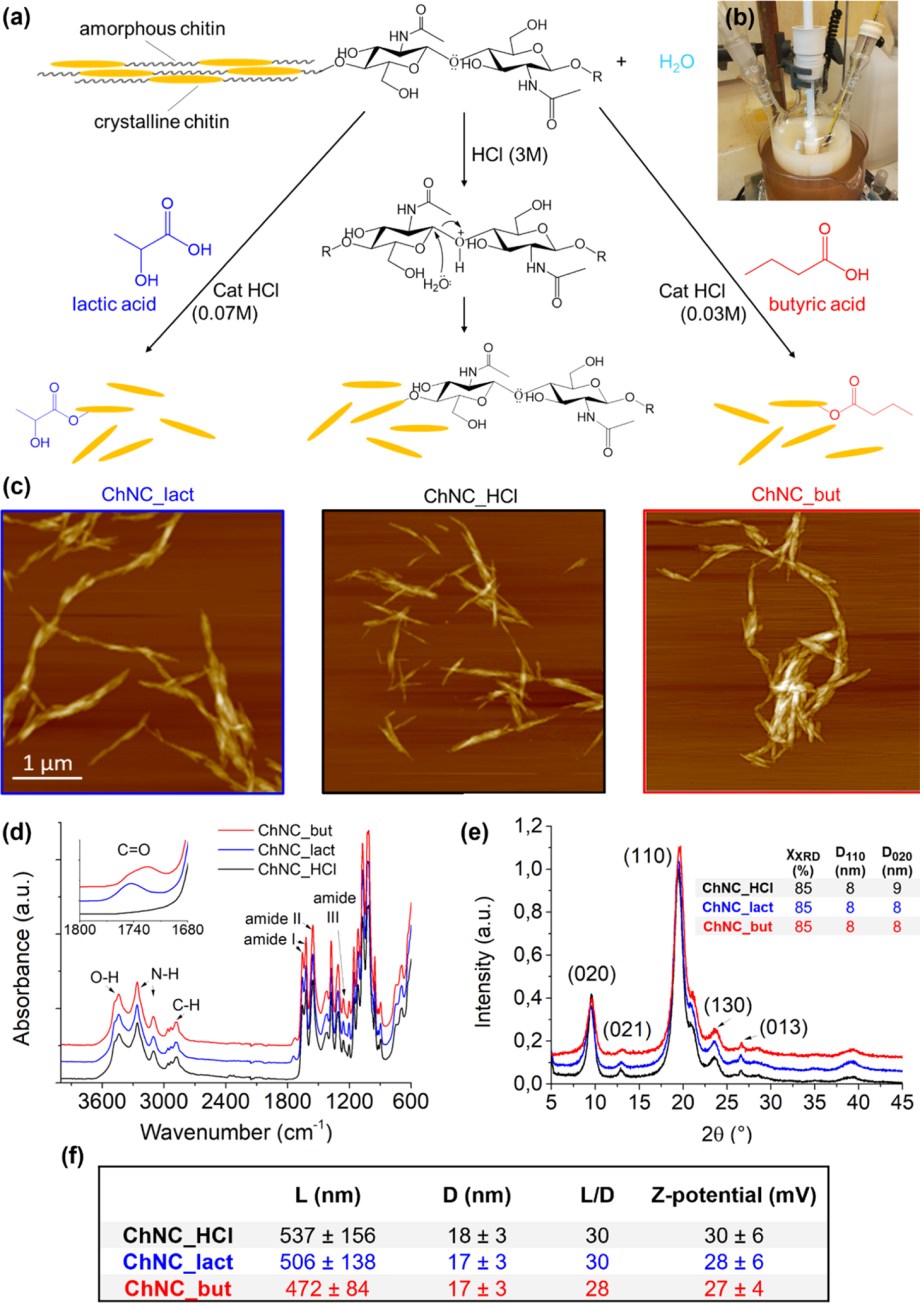
Schematic representation of ChNC extraction (a) and the system built for it (b). The crystalline regions of chitin are reported as yellow and needle-shaped, while the amorphous regions correspond to the black wavy lines. The central path shows the classical acid hydrolysis of raw chitin with hydrochloric acid 3 M with the obtained nanocrystals indicated as ChNC_HCl. The paths on the sides report the extraction through one-pot acid hydrolysis/Fischer esterification: in presence of lactic acid and HCl 0.07 M (on the left) and butyric acid and HCl 0.03 M (on the right). ChNCs lactate-modified and butyrate-modified are called ChNC_lact and ChNC_but, respectively. AFM analysis (c) shows the needle-shaped morphology. FT-IR spectra (d) are reported with the onset zoomed on the signal corresponding to the carboxyl stretching of the ester group. (e) XRD diffractograms are reported and the table contains the crystallinity index (CI) obtained after deconvolution (see Supporting Information) and the crystallite dimensions calculated with the Scherrer equation for the crystalline faces 110 and 020. (f) Table at the bottom reports the length (*L*) and diameter (*D*) measured from AFM and STEM analysis. The aspect ratio (*L*/*D*) and the ζ potential are also reported.

Appropriately tuning the extraction parameters, controlled ChNC dimensions and the functionalization degree, has been designed and successfully achieved to highlight only the effect of surface modification on inter-nanocrystal interactions. A quantitative determination of the degree of surface modification of ChNCs has been assessed by solid-state NMR spectroscopy, supported by measured morphological features.

Nematic structures of ChNC water dispersions were observed under shear with the rheo-PLI technique (rheology coupled with polarize light imaging), directly correlating optical and rheological properties.^39^ This technique allowed the drafting of a phase diagram of nanocrystal supramolecular interactions as a function of the topochemical moieties and their colloidal concentration.

As a proof of concept, we have targeted Pickering emulsions as a relevant application that benefits from exploiting individualized nanocrystals. Indeed, an emulsion stabilization takes place at the interface between immiscible phases where nanoparticles migrate.^40^ The higher the interface mediated by the nanoparticles, the lower the interphase surface tension that hinders coalescence.^40^ That is why individualized ChNCs are the efficient stabilizers for Pickering emulsions.^21−24^ It is worth to note that the successful examples reported in the literature are obtained employing never dried ChNCs. To highlight the effect of the topochemical modification to preserve ChNC individualization, we have prepared our Pickering emulsions from lyophilized ChNCs. Furthermore, to our knowledge, the effect of ChNC surface chemistry in Pickering emulsions has not been investigated yet.

Our results demonstrate the relationship between topochemical modification and ChNC self-assembly. As a consequence, topochemical modification enables effective use of regenerated modified ChNCs from the dried state. This possibility to use lyophilized ChNCs instead of their diluted water suspensions (above 90%), where the individualized nanoparticles are relevant, paves the way to broaden their application, impacting their commercialization, for example, the volume for their transport and stock. In addition, we suggested rheo-PLI as an effective technique for discerning between individualized nanocrystals or their supramolecular assemblies.

## Results and Discussion

### One-Pot Acid Hydrolysis/Fisher Esterification as a Green Efficient Method for Preparation and Surface Modification of Size-Controlled ChNCs

Chitin and cellulose are polysaccharides characterized by glycosidic linkages and hydroxyl groups present in each glucose-derived monomeric unit (Figure 1 and Scheme S1). Both biopolymers self-organize in crystalline nanodomains surrounded by amorphous regions; therefore, a one-pot acid hydrolysis/Fischer esterification (scheme in Figure 1) is proposed as a green method to obtain ChNCs in light of previous successful isolation of CNCs.^35−37^

Hydrogen ions from HCl dissociation have a double action: (i) they can hydrolyze the glycosidic linkages of the amorphous and more accessible chitin regions; and (ii) they can catalyze the reaction between the carboxylic group of the organic acid and the exposed hydroxyl groups on the so-isolated nanocrystals’ surface. As a result, chitin is concurrently hydrolyzed and esterified by using a catalytic amount of HCl, yielding surface-modified nanocrystals in one single step (Figure 1 and Scheme S2). It is worth noting here that the extraction method developed in presence of renewable organic acids improves the sustainability and the “green” character of the classical water-based acid hydrolysis because it significantly reduces HCl concentration by about a factor of hundred times. As a proof of concept, renewable lactic and butyric acids were selected as short-chain organic acids with different acid strengths (acid dissociation constants, p*K*_a_, are 3.7 and 4.8, respectively) for the validation of the one-step hydrolysis and esterification of chitin. As a control, ChNCs (ChNCs_HCl) have been extracted through conventional acid hydrolysis (HCl 3 M).^41−43^

The analysis of the atomic force microscopy (AFM) micrographs discloses typical needle-shaped morphology of ChNCs (Figure 1, bottom images), regardless of the extraction method, confirming the efficiency of the procedure in one single step proposed.

The yield obtained for esterified nanocrystals is between 40 and 60% under all the extraction conditions tested. This yield is remarkably higher compared to that of other green methods of ChNC preparation proposed in the literature,^44^ and it relates to the catalytic effect of HCl. Even compared with CNCs obtained with the same method, the yield obtained is higher, suggesting greater sensitivity of chitin to hydrolysis. Moreover, surface-decorated ChNCs are obtained in one single step, reducing consistently the use of large excess in chemicals and the purification steps.

After the validation of the extraction of esterified ChNCs, an optimization of the process control was carried out to obtain butyrate (ChNC_but) and lactate (ChNC_lact) surface-modified nanocrystals with homogeneous properties (size, acetylation, and modification degree). Furthermore, the control of the reaction for the production of comparable ChNCs with only different surface chemistry enables the study of their supramolecular organization in the colloidal state, that is, the effect of different surface moieties on the inter-nanocrystals’ interactions.

Different reaction conditions have been tested for HCl hydrolysis and the one-pot method (Table S1) playing on the timing and HCl concentration to obtain nanocrystals with comparable dimensions and aspect ratios (Figure 1f).

Two methods have been used to select the reaction parameters satisfying the required comparable size. The thickness of nanocrystals was measured by AFM analysis (Figures 1c and S1), and the length was measured from micrographs obtained through transmission electron microscopy (TEM) and scanning electron microscopy in the transmission mode (STEM) (Figure S2). Nanocrystals with an aspect ratio around 30 (length ≈ 500 nm and thickness ≈ 17 nm) have been considered suitable for colloidal structuring studies. Therefore, their structural and surface features were further investigated. For the sake of clarity, the selected samples ChNC_but_0.03-3 and ChNC_lact_0.07-3 (see Table S1), where the first number refers to the concentration of HCl (0.07 or 0.03) and the second one (3) refers to the hours of reaction, will be named ChNC_but and ChNC_lact, respectively.

**Figure 2:**
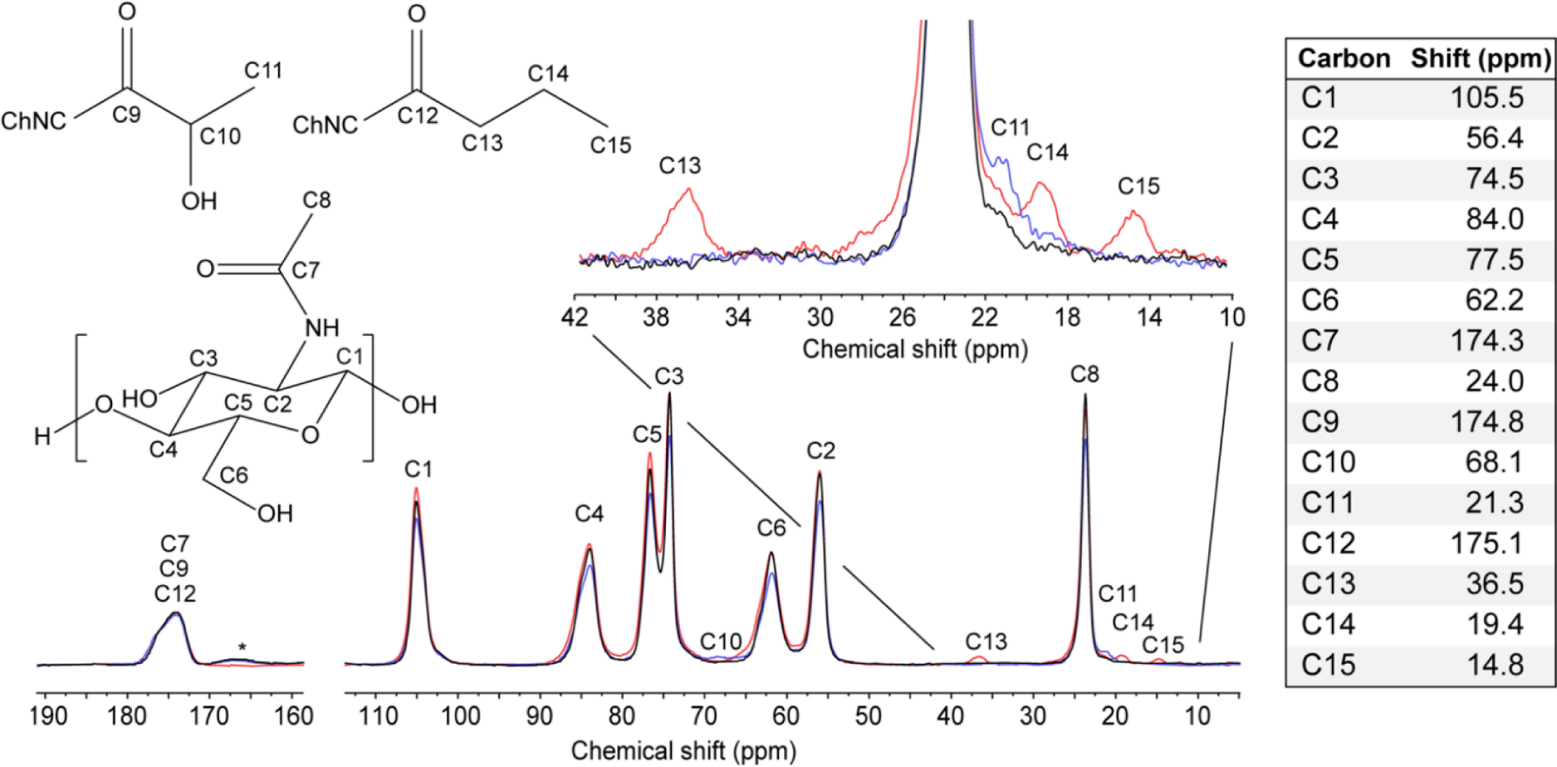
Molecular structure of the chitin repeating unit and the lactate and butyrate modifications with all carbons numbered (top left) and relative chemical shifts (table); ChNC denotes the oxygen bridge to C6, or possibly C3, of the chitin; CP/MAS ^13^C NMR spectra of ChNC_HCl (black), ChNC_lact (blue), and ChNC_but (red) with carbons assigned (bottom spectrum); upfield regions of the CP/MAS ^13^C NMR spectra of ChNC_HCl (black), ChNC_lact (blue), and ChNC_but (red), emphasizing the signals assigned to the chemical modifications.

Optical spectroscopic analysis of the different ChNCs shows evidence of their surface modification in presence of an organic acid (Figure 2). Infrared spectra of extracted ChNCs exhibit signals characteristic of native chitin (Figure S3), in particular at around 3444 cm^–1^ (O–H stretching vibration), 3257 and 3103 cm^–1^ (N–H stretching), 2885 cm^–1^ (CH_2_ and CH_3_ stretching), 1654 and 1621 cm^–1^ (amide I bands), 1554 cm^–1^ (amide II bands), and 1260 cm^–1^ (amide III).^6^ The Fourier transform infrared (FT-IR) spectra of ChNC_but and ChNC_lact are characterized by the appearance of the peak related to C=O stretching at around 1730 cm^–1^, demonstrating successful esterification of the nanocrystals.^36,45^

The surface modification did not affect the crystalline lattice of chitin, as proved by the X-ray diffraction (XRD) analysis. XRD diffractograms (Figures 2 and S4) of ChNC_HCl, ChNC_but, and ChNC_lact show diffraction peaks at 2θ = 9.3° (020), 12.6° (021), 19.3° (110), 23.1° (130), and 26.3° (013), in agreement with the native chitin (Figure S4) and corresponding to the typical crystal patterns of α-chitin.^6^ The degree of crystallinity has been calculated as the ratio between the sum of crystal peak integrals (obtained through deconvolution, Figure S5) and the integral of the whole diffractogram. Native chitin has a crystallinity of 80%, which increases to 85% after HCl hydrolysis, underlining an efficient removal of the amorphous regions during the nanocrystals’ extraction. Similar values are assessed for both the esterified ChNCs, confirming that the one-pot Fisher esterification catalyzed by HCl leads to a successful extraction comparable with the classical acid hydrolysis.

According to the orthorhombic geometry of α-chitin, the 001 set of planes corresponds to the longitudinal axis of the nanocrystals, so 020 and 110 planes correspond to the transversal axes. If the hydrolysis starts to peel off the crystalline part of chitin, a decrease in the crystallite dimensions should be seen. The Scherrer equation was used to estimate crystallite size obtaining *ca.* 8 nm for the plane 020 and 110 for all the nanocrystals. This agrees with the values found in the literature.^46^ It is worth noting that the crystallite size estimated from XRD analysis reflects only the crystalline domains, which together with the amorphous domains contribute to the ChNC diameter measured by AFM. Therefore, it is not surprising that the AFM diameter assessed is larger than the XRD crystallite size. This comparison demonstrates that the designed hydrolysis compromised only the amorphous regions of chitin, so the designed one-pot hydrolysis/Fisher esterification preserved chitin-inherent crystalline regions.

### Topochemical Feature Assessments

To selectively highlight the effect of different surface chemistry on colloidal dispersions, all other parameters influencing the rheological behavior and supramolecular structuring were carefully minimized. The successful modification (FT-IR, Figure 1b) was proved qualitatively and, the comparable chitin nanocrystal size was proved quantitatively (AFM, Figures 1c,f and S1, and TEM/STEM, Figure S2). However, both AFM and TEM/STEM morphologies show the nanocrystals after different drying performed for the sample preparation, that is*,* they do not provide information about their dispersion. Also, the pH and ζ potential of ChNC water dispersions were measured, and the results confirm that all ChNCs showed comparable values of ζ potential of *ca.* 28 mV (Figure 1f) while maintaining a constant pH at 5.8, corresponding to pure deionized (DI) water. It is worth to note that the suspensions in DI water, without adjustment of pH or ionic strength, are expected to show relatively low ζ-potential values compared to those of other reported studies because of low amount of ions (already in DI water) and lower amount of protonated amines (p*K*_a_ ≈ 6.3).^15,16^ Moreover, ChNCs form lyophilic water colloids because of the numerous hydrogen bonds with the dispersive medium. Therefore, the assessed ζ-potential lower than 30 mV does not imply an incipient instability, as for other lyophobic colloids.^47^

Fischer esterification and simultaneous hydrolysis could lead to a different degree of modification and deacetylation.^15^ If the acidic conditions which lead to the hydrolysis of amorphous chitin persist, the amide groups could be reduced, leading to the formation of amines, that is, impacting the acetylation degree. At the same time, the Fischer esterification catalyzed by HCl has different kinetics depending on the organic acid. Dedicated reaction parameters are required for the sake of obtaining a comparable degree of modification. A detailed nano-structural and chemical characterization of the ChNC topochemical features is mandatory to quantitatively assess the degree of acetylation (DA) and modification and to unravel the relationship between reaction conditions–nanostructure–physical properties. Therefore, solid-state nuclear magnetic resonance (NMR) spectroscopy was selected to quantitatively determine the DA, and the degree of substitution (DS) for lactate and butyrate modifications. Figure 2 shows the molecular structure of the chitin repeating unit, the lactate and butyrate modifications, and the solid-state cross-polarization magic angle spinning carbon-13 (SS CP/MAS ^13^C) NMR spectra of ChNC_HCl (black), ChNC_lact (blue), and ChNC_but (red), with all carbons assigned. The chitin signals were assigned using chemical shifts from the literature,^48^ and the assignments, including those for the lactate and butyrate modifications, are summarized in the table of Figure 2.

The CP/MAS pulse sequence is widely used as a tool for structural analysis but it is not inherently quantitative due to the difference in effective magnetic transfer to carbons coupled with a different number of protons or with different molecular dynamics.^49^ However, with the contact time being chosen carefully, the pulse sequence could enable to provide quantitative results. Contact times of around 1 ms have previously been used to quantitatively determine DA^50,51^ and DS of modifications,^52^ (Table 1, and details in Experimental Section).

**Table 1:** DA, DS, DS on Surface (DS_surf_), and SF for All Samples^a^

sample	DA (%)	DS_lact (%)	DS_but (%)	DS_surf__lact (%)	DS_surf__but (%)	SF (%)
ChNC_HCl	100					16
ChNC_lact	97	4.7 ± 0.4		30 ± 3		16
ChNC_but	94		5.6 ± 0.4		36 ± 3	16

a DA and SF were determined with a precision of 5%.^51,53^

In Figure 2, the region of the CP/MAS ^13^C NMR spectra that includes the signals assigned to chemical modifications, are shown for ChNC_HCl, ChNC_lact, and ChNC_but.

DS for lactic and butyric acid modifications could be calculated by comparing the integrals related to the modifications with those of the chitin backbone integrals.

The DS value (Table1) gives the amount of modification compared to all chitin in the sample. However, in the case of modified ChNCs, the modification will only occur on the surface, and hence a more informative value would be to consider only the accessible chitin moieties and calculate the DS on the surface, DS_surf_. Parameter DS_surf_ can be calculated by knowing the surface fraction (SF) of the chitin moieties *a priori*, that is, the degree of chitin located on the crystallite surface in relation to the total amount of chitin. For CNCs, SF has previously been obtained from solid-state NMR spectroscopy measurements by deconvolution of the cellulose C4 region.^53^ For CNCs, the NMR C4 signals assigned to the accessible surface are distinguished from those assigned to both inaccessible amorphous and crystalline materials. Hence, the SF and CI can be obtained by spectral analysis. Recently, solid-state NMR spectroscopy has also been used to calculate the CI of chitin samples.^54^ The spectrum of chitin is however much less resolved compared to that of cellulose, and any specific accessible surface signals are not provided by the reported method, solely the crystalline and amorphous parts as such. However, in this study, the SF could be obtained using the cross-section dimensions of the ChNC rods obtained from AFM studies.

The reaction design enabled comparable SFs (from AFM measurements, Figure 1c), which have been used in support of the characterization of the surface topochemical features. The DA of the different ChNCs is slightly affected by the different reaction designs. The weaker the acid (p*K*_a HCl_ < 1, p*K*_a lactic acid_ = 3.7, and p*K*_a butyric acid_ = 4.8), the lower the acetylation degree. Our results are consistent with the assumption that the esterification occurs only on hydroxyl functionalities and not after amidation of free amines. The assessment of the degree of surface modification and acetylation for the selected samples confirmed that the reaction design was suitable to obtain quantitatively comparable topochemical features.

### Rheo-PLI of Liquid Crystalline-like Structures

In the colloidal state, nematic structuring has been demonstrated for ChNCs, which is affected by the inter-nanocrystal interactions.^15,16,19,33^ However, to our knowledge, the effect of surface chemistry on colloidal properties in ChNCs has not been explored yet. Therefore, once verified for the different ChNCs prepared that the aspect ratio and degree of surface modification were comparable, the rheological properties of their water colloidal dispersion were investigated. Furthermore, a polarized light visualization setup has been coupled with a rheometer (rheo-PLI) to correlate ChNC nematic structures and topochemical moieties, in our knowledge also for the first time.

Rheology can be a valuable tool to assess the self-assembly of nanostructures and their dynamic behavior in suspension.^39^ First, we briefly discuss the ChNC phase behavior and the influence of ChNC size thereon, whereafter we focus on the influence of topochemical modifications on their nematic structuring. Similar to other liquid crystalline systems, with increasing concentration, ChNC suspension develops from an isotropic phase to a biphasic phase consisting of both isotropic domains and chiral nematic/nematic domains. With diminishing isotropic domains, the system transitions to a liquid crystalline phase and finally to a glassy state.^55^

**Figure 3:**
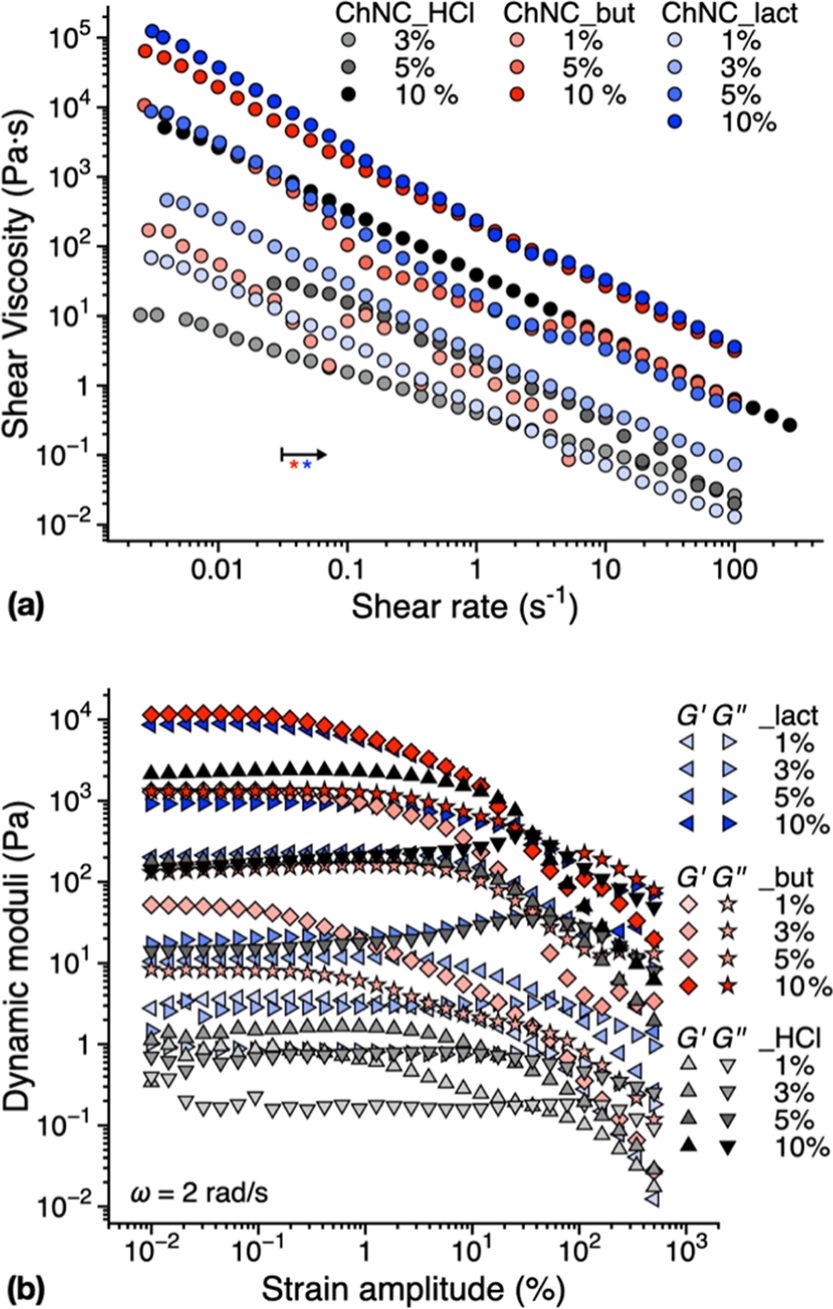
(a) Steady shear viscosity functions and (b) dynamic moduli from strain sweep tests showing the influence of topochemical modifications and concentration. In (a), the arrow approximates the shear rates above which ChNC_but and ChNC_lact data are affected by bubble inclusions and phase separation.

**Figure 4:**
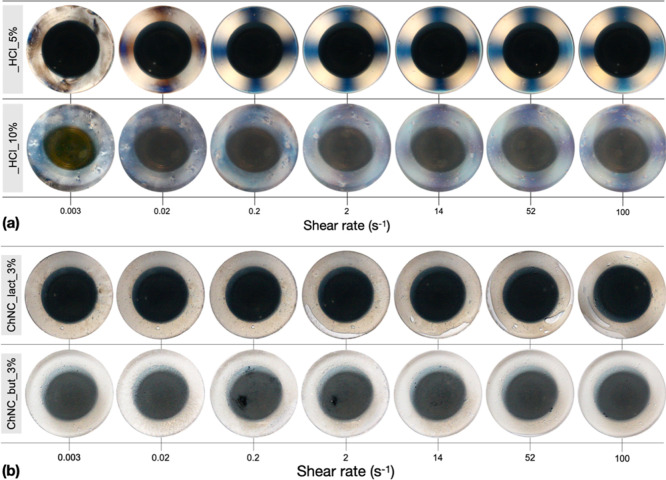
PLI still frames showing the circumferential birefringence patterns at selected shear rates from the steady shear tests: (a) ChNC_HCl and (b) ChNC_lact and ChNC_but at 3 wt %.

By combining steady shear data, Figures 3a and S6, with simultaneous PLI, Figures 4 and S7, the phase behavior can be estimated.^56^ For concentrations ≤3 wt %, both sizes of ChNCs tested (regular, ChNC_HCl, and small, ChNC_HCl_s) exhibit very weak birefringence patterns at the start while at high shear rates, a colored Maltese cross pattern was recorded, indicating the presence of chiral nematic/nematic domains predominantly oriented in the flow direction.^56,57^ In addition, the steady shear viscosity functions disclose a three-region behavior, particularly for the ChNC_HCl_s (Figure S6). Although such a behavior is characteristic of liquid crystalline systems, its assignment to a particular phase remains challenging.^39^ The presence of large isochromatic areas observable for ChNC_HCl_s at the beginning of the tests suggests a liquid crystalline phase state, with a weak three-region behavior for 3–5 wt %. On increasing the concentration, the systems appear to approach the glassy state independent of the size and aspect ratio of ChNC_HCl, as evidenced by the diminishing colors (compare especially concentrations 5.7 and 10 wt % in Figures 4 and S7). The higher size/aspect ratio of ChNC_HCl can be mainly seen on the comparatively early onset of the gel-like behavior in dynamic strain sweep tests (Figure S6b) and by reaching flow-scale orientational order, that is, the onset of the Maltese cross pattern, at lower shear rates (Figures 4 and S7).

Steady shear data for ChNC_lact and ChNC_but appear more challenging to analyze due to significant anomalies in their viscosity functions. By anomalies, we refer to discontinuities in the viscosity functions such as for ChNC_but 1% at *ca.* 0.05 and 5 s^–1^ and for ChNC_HCl 5% around 20 s^–1^ or the apparent distinct shear rate regions, such as ChNC_but and _lact 5% (slightly visible also at 10%) between approximately 0.1 to 6 s^–1^. However, PLI visualizations reveal that both samples exhibit bubble inclusions at shear rates as low as 10^–2^ s^–1^, and an anomalous behavior can be seen thereon (Figures 4b and S8). Also, the sample ChNC_lact 1% shows unattended phase separation that is not present at higher concentrations and which could contribute to the anomalous behavior. In addition, both samples did not disclose significant birefringence patterns, and no Maltese cross was observed within the highest shear rates in the study. The shear dynamic moduli from strain sweep tests of ChNC_HCl exhibit a gel-like behavior already at 1 wt % (Figure 3b). Overall, both ChNC_but and ChNC_lact infer higher interactions at low shear strain amplitudes (higher dynamic moduli) compared to ChNC_HCl, in agreement with the steady shear data. Above 3 wt %, ChNC_HCl and ChNC_lact show weak strain overshoot (increasing *G*″), which has been related to the jamming of the microstructure before significant microstructural changes.^58^ Interestingly, a weak strain overshoot can be observed for ChNC_lact up to 5 wt % but not for ChNC_but. It can be here inferred, that the rheological behavior of ChNC_lact is more similar to that of ChNC_HCl due to the hydroxyl group reintroduction together with the lactate moieties on the nanocrystals’ surface, favoring their inter-nanocrystal interactions.^15,38^ However, being a weakly nonlinear behavior, this could also be attributed to the artifacts observed in the steady shear.

**Figure 5:**
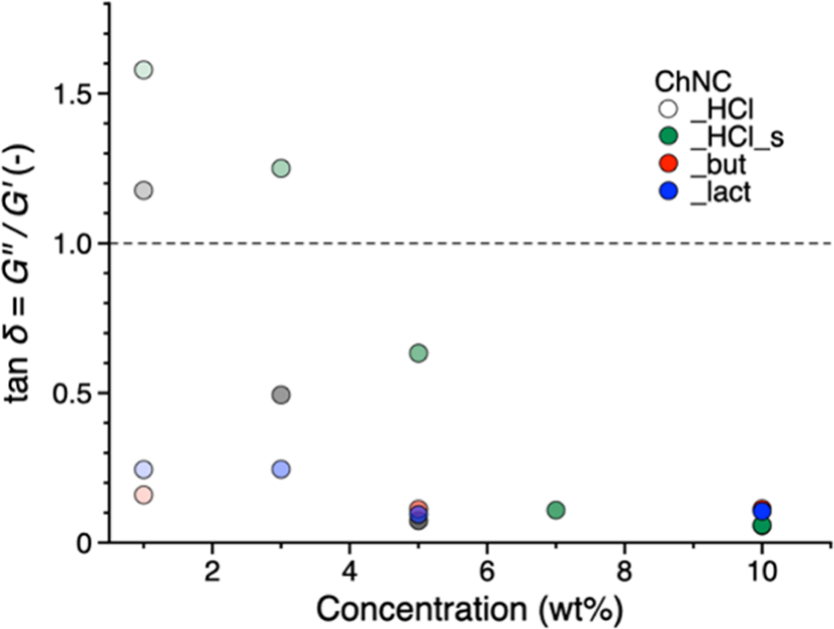
Loss tangent, tan δ = *G*″/*G*′, as a function of concentration for all ChNC samples studied.

**Figure 6:**
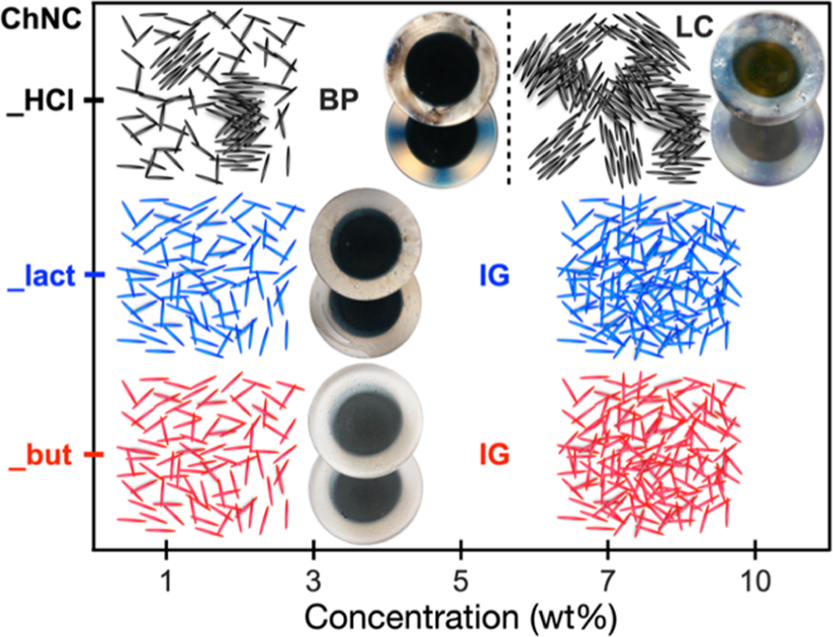
Phase diagram summarizing the phase behavior of the ChNCs investigated: BP—biphasic, LC—liquid crystalline, and IG—isotropic gel.

Overall, in the linear viscoelastic limit, both ChNC_lact and ChNC_but show higher gel strength at low concentrations, as quantified by the loss tangent, tan δ = *G*″/*G*′ (Figure 5). All the colloids based on modified ChNCs show a higher elasticity of about 1 order of magnitude larger than unmodified ones (Figure 3b). To obtain the elastic moduli and viscosity of the 10 wt % ChNC_HCl, the quantity of modified nanocrystals required is halved. Above 5 wt %, all samples approach a limiting loss tangent, demonstrating the prevalent elastic character of the samples, typical of a glassy state. We can therefore conclude that in terms of the phase behavior and flow, in the concentration range investigated, both ChNC_lact and ChNC_but start as isotropic gels likely reaching a glassy state at the higher end of the concentration range, Figure 6.^59^ These results suggest the use of modified ChNCs in applications where a high gel strength is desired, for example, pharmaceutical and cosmetic applications. It is worth to note that the studied water suspensions have shown to be stable up to 10 wt % in a gel form that can be easily re-diluted.

Surface modification distinctly affects the supramolecular organization of ChNCs, hindering their nematic structuring, regardless of the specific moieties, as summarized on the phase diagram in Figure 6. Topochemical modification disturbs the inter-nanocrystals hydrogen bonds, exposing changed surface moieties to the surrounding water. As a consequence, the gelation of esterified ChNCs occurs already at lower ChNC concentration (1 wt %), pointing out a prevalent ChNC surface interaction with the surrounding media. Our results demonstrate that ChNC individualization can be obtained by controlled chemical modification (about 5% DS, corresponding to about 30% of the surface, Table 1), maximizing the ChNC surface on the nanoscale. Both in the isotropic and the glassy states, compared with liquid crystalline unmodified nanocrystals, the modified ones can provide chemical moieties more available for further modification or interactions with biological molecules and therefore more suitable for applications in which individualization of the nanomaterials is of interest.

### Proof of Concept: Chitin Nanocrystals as Pickering Emulsion Stabilizers

To exemplify an application in which individualization of the nanoparticles is crucial, Pickering emulsions have been prepared using sunflower oil in DI water (1:10)^24^ with the addition of 0.85 w/v % chitin nanocrystals previously lyophilized.

**Figure 7:**
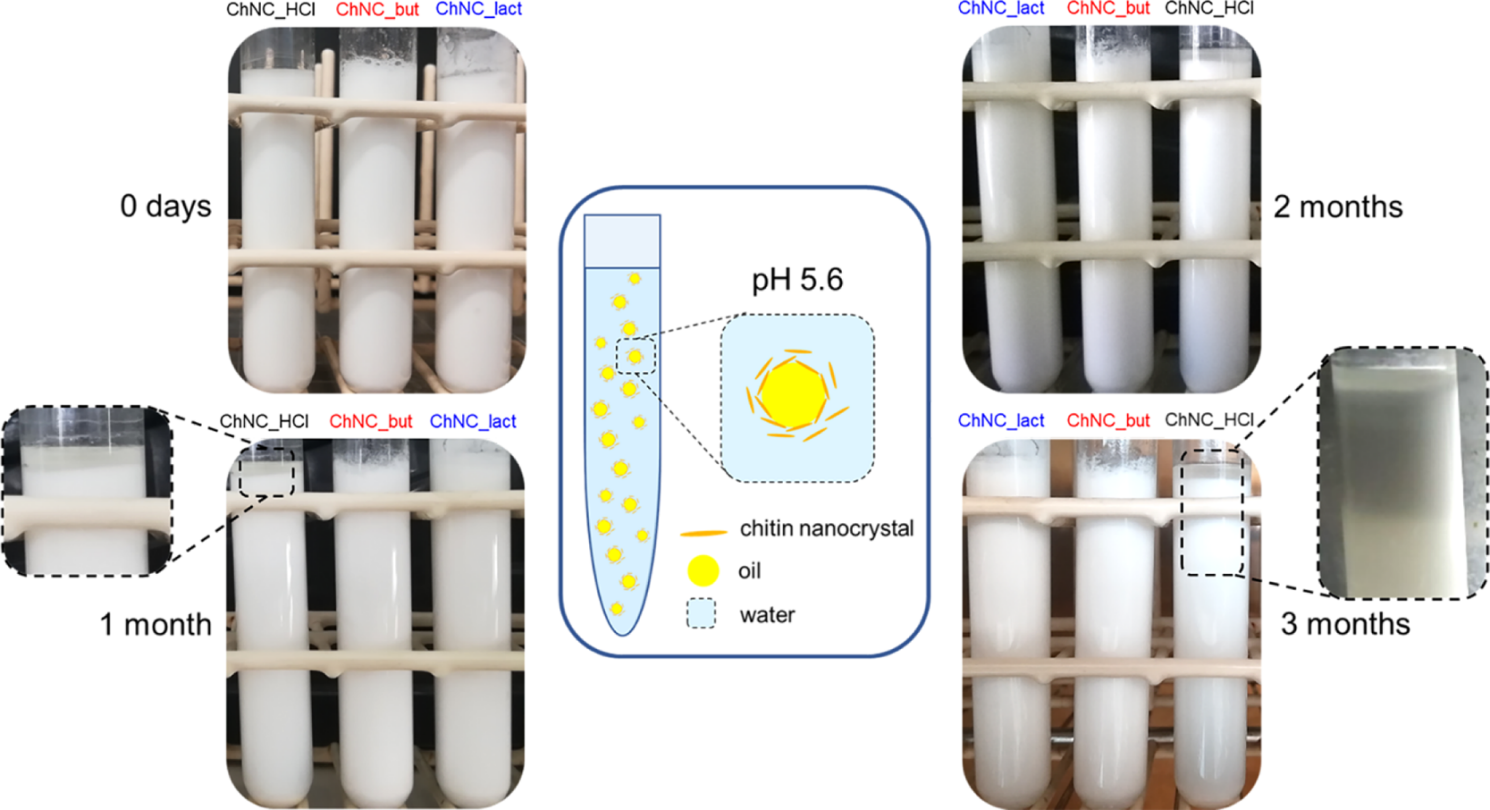
Pickering emulsion obtained with the addition of 0.85 wt % lyophilized ChNCs to a mixture of water and corn oil at 10 wt % after 1′40″ sonication with a probe. We have used DI water obtaining a final pH of 5.6. In the middle, a schematic representation of a Pickering emulsion is reported. The emulsions obtained with ChNC_HCl, ChNC_but, and ChNC_lact are shown just after the emulsification (0 days) and after 1, 2, and 3 months. The insets zoom on the creaming effect and phase separation of the emulsion obtained with unmodified ChNC_HCl.

All the nanocrystals form Pickering emulsions as it is clear from the visual aspect of the prepared samples (Figure 7). For the sake of comparison, a control emulsion of oil in water without any ChNCs has been prepared using the same preparation procedure. This control emulsion undergoes an incipient phase separation starting right after the sample sonication (Figure S9). After 1 month, only the emulsion containing ChNC_HCl shows a creaming (oil on the top of the emulsion), indicating incipient phase separation, while both the emulsions containing modified ChNCs still appear stable. The impact of the increased hydrophobic character and/or individualization of modified nanocrystals becomes even more evident after 3 months (Figure 7, bottom-right corner). These results can be ascribed to both the relatively more hydrophobic moieties (butyrate and lactate) on the ChNC surfaces and the higher individualization of modified ChNCs, compared to ChNC_HCl, after regeneration from the lyophilized state. Although each lactate moiety reintroduces a hydroxyl group on the ChNC surface, the steric hindrance of the ester function is predominant, and it prevents the formation of irreversible aggregates. It is worth to note the relevance of our results in the frame of the literature. Previous studies have reported successfully stabilized emulsion by using never dried ChNCs from water dispersion, while our results highlight that stable Pickering emulsion can be effectively prepared from the modified ChNCs redispersed after lyophilization.

The two possible mechanisms of the observed stabilization, individualization, and patchy hydrophobic surface point chemical modification as a successful strategy for improved performance of ChNCs in Pickering emulsions. This preliminary test confirms how a well-designed topochemistry can broaden the application field of ChNCs because of their capacity to stabilize hydrophobic components in water, which is of great interest in various applications, for example, in cosmetic, food, and pharmaceutical.

## Conclusions

Decoration of ChNCs with butyrate or lactate moieties has been successfully obtained during a sustainable and green one-pot acid hydrolysis/Fischer esterification in water. The controlled reaction enabled the preparation of nanocrystals with a comparable size and degree of modification, which differ only for the topochemical moieties. Rheo-PLI analysis unraveled how topochemical modification of ChNCs affects their self-assembly in water colloidal suspension. Both lactate and butyrate moieties hinder the organization of ChNCs in liquid crystals in favor of their individualization. Because topochemical modification disturbs the inter-nanocrystal interactions, it exposes surface moieties to the surrounding water, lowering the gelation point (already at 1 wt %), in comparison to unmodified ChNCs. In other words, ChNC–media interactions are strengthened by the individualization of esterified ChNCs.

Finally, we have prepared Pickering emulsions using ChNCs from the dry state as stabilizers. Our results showed a stability over 3 months of emulsions prepared with esterified ChNCs, for both lactate and butyrate moieties, while an incipient phase separation was observed in the emulsion containing unmodified ChNCs. Thus, chemical modification of ChNCs not only improved the stabilization of oil-in-water emulsions, thanks to their surface patchy hydrophobic/hydrophilic character (in ratio ≈30/70), but also demonstrated to be an efficient strategy to resuspend lyophilized nanocrystals. Our result paves the way for the use of dried modified ChNCs for applications where their individualization is required, overcoming the limitation of ChNC widespread use due to the large amount of water needed for their transport and stock.

Thanks to their demonstrated enhanced individualization upon drying, these modified ChNCs are under evaluation for the preparation of nanocomposites, where well-dispersed ChNCs would provide improved mechanical performance.

## Experimental Section

### Materials

Chitin from shrimp shells (practical grade, code C9213) was purchased from Sigma-Aldrich; hydrochloric acid 37% of the technical grade was purchased from PanReac AppliChem (ITW Reagents); lactic acid (85% FCC, code W261106-1KG-K) was purchased from Sigma-Aldrich, and butyric acid (for synthesis, code 8.00457.2500) was obtained from Merck Millipore. Nylon membrane filters (pore size 0.22 μm) were purchased from FilterLab.

### ChNC Preparation

10 g of chitin were soaked in 225 mL of DI water in a three-neck round-bottom flask and the temperature was risen to 100 °C, keeping the suspension under mechanical stirring with the help of an overhead stirrer (IKA-Werke GmbH & Co. KG, Germany) equipped with a Teflon centrifugal stirrer shaft (Thermo Fisher Scientific Inc., Sweden) (500 rpm). Then, 75 mL of HCl 12 M was added. After the addition of all the reagents, the chitin concentration was 30 mL/g and HCl final concentration was 3 M. The acid hydrolysis was stopped after 90 min. To increase the pH to 6, the mixture was filtered six times with a nylon membrane with pores of 0.22 μm using high-pressure filtration equipment. Each time, the cake collected from the filter was resuspended in water with the help of T18 digital Ultra Turrax equipped with a S 18 N—19 G dispersing tool (IKA-Werke GmbH & Co. KG, Germany).

To collect the nanocrystals, the suspension was centrifugated at room temperature (5 min, 3800 rpm), recovering the opalescent supernatant and resuspending the precipitate containing unhydrolyzed chitin. The procedure was repeated 10 times, and the collected supernatants were unified and filtered with a fritted glass filter with porosity 3 to eliminate any possible residue of microchitin. The final sample was stored in a fridge at 6 °C.

### Functionalized ChNC Preparation (One-Pot Acid Hydrolysis/Fischer Esterification)

5 g of chitin was soaked overnight in a mixture of DI water and the desired organic acid (lactic or butyric acid) at room temperature. Then, the temperature was risen until reflux (116 °C for lactic acid and 107 °C for butyric acid) under mechanical agitation (500 rpm) and a catalytic quantity of HCl 12 M was added to reach the desired final concentration. The final concentration of chitin was 0.04 mg/mL. The reaction was stopped after 3 or 5 h, and the chitin was separated from the rest through centrifugation (8000 rpm, 15 min, and 5–10 °C reached in a freezer). Each mixture was redispersed in DI water and filtered four times (with a nylon membrane with pores of 0.45 μm using high-pressure filtration equipment) to reach pH = 6, redispersing the cake on the filter in clean DI water using Ultra Turrax. Then, nanocrystals were collected and separated from unhydrolyzed chitin through centrifugation (3800 rpm, 5 min, RT), each time collecting the supernatant and resuspending the precipitate with Ultra Turrax. For each sample, this procedure was repeated 10 times. In the end, all the collected supernatants were filtered with a fritted glass filter (porosity 3) to obtain a pure sample of ChNCs.

### Pickering Emulsions

Pickering emulsions have been prepared using sunflower oil in DI water (1:10)^24^ with an addition of 0.85 w/v % ChNCs. ChNCs were redispersed in the water fraction through mechanical mixing with the help of a mini vortex mixer VM-3000 (VWR International) before the addition of oil. An ultrasound probe (130 W ultrasonic processor VCX 130, 6 mm probe, frequency 20 kHz, Vibra-Cell, Sonics) was used to emulsify each system applying a power of 30 W for 1 min and 40 s and avoiding overheating with the help of an ice bath. We have used DI water obtaining a final pH of 5.6.

### Characterization

The yield of each preparation was calculated as the ratio between the weight of raw chitin and the weight of recovered nanocrystals after lyophilization.

### Attenuated Total Reflectance FT-IR

It was carried out using a Bruker Tensor 17 spectrometer.

### ζ Potential

The measurements were performed with a Zetasizer Nano ZSP (Malvern) system on ChNC water suspension at a concentration of 0.01 mg/mL.

### pH Measurement

The pH was measured with the help of an instrument 827 pH lab meter (Metrohm).

### Morphological Analysis

The morphology of nanocrystals was studied with three techniques: TEM analysis was carried out with an apparatus Philips CM200 at an acceleration voltage of 20 kV; STEM analysis was carried out with an apparatus HITACHI SU8020 in the transmission mode; AFM measurements were performed on dried suspensions of 0.05 mg/mL in chitin content for ChNCs in the tapping mode in air using a Digital Instrument Dimension 3000 large sample with a standard silicon cantilever with a type G scanner (Digital Instruments Inc.). Measurements of the diameter of ChNCs were performed by measuring the nanocrystal height using NanoScope software. TEM and STEM analyses were used to measure the length of the nanocrystals while AFM was used for the diameter. The aspect ratio was calculated from the morphological analysis on 100 different individualized ChNCs.

### XRD

It was assessed on a Panalytical Empyrean diffractometer with an area detector operating under Cu Kα (1.5418 Å) radiation (40 kV, 40 mA). The crystallinity was assessed as the areas of the crystalline diffraction peaks to the total area under the curve between 2θ = 10 and 50°. The crystallinity of ChNCs and bionanocomposites was determined based on the Rietveld–Ruland approach
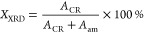
where *A*_CR_ is the area for crystalline peaks and *A*_am_ is the area for amorphous peaks. Deconvolution of XRD diffractograms has been performed with PeakFit v4.2 software (Jandel Scientific Software) and using Gaussian–Lorentzian line shapes for the fitting (see Figure S5). The amorphous contribution was individuated in two halos, the main one at around 21.5° (related to intermolecular scattering) and a smaller one around 41.5° (related to intramolecular scattering).^60^

The crystallite sizes of 002 lattice planes were estimated by using the Scherrer equation^61,62^
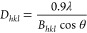
where *D*_*hkl*_ is crystallite size in the direction normal to the *hkl* lattice planes, λ is the radiation wavelength (1.54 Å), and *B*_*hkl*_ is the full width at half-maximum in radians of the corresponding *hkl* lattice planes.

### Solid-State NMR

SS CP/MAS ^13^C NMR experiments were performed with a Varian Inova-600 spectrometer operating at 14.7 T and equipped with a 3.2 mm solid-state MAS probe. Measurements were conducted at 298 K with a MAS spinning rate of 15 kHz. A CP/MAS ^13^C NMR pulse sequence with a SPINAL-64 decoupling sequence was used. Acquisition parameters included a 2.9 ms ^1^H pulse, a 900 ms CP-contact time, 25 ms acquisition time, 4 s recycle delay, and 16 384 scans. The chemical shifts were referenced to adamantane with the CH_2_ signal being set to 38.48 ppm. The DA^50,51^ and DS of modifications^52^ have been quantitatively determined as follows:

The DA has been obtained by comparing the integral of the acetyl methyl carbon C8 (Figure 2) with the average integral *I*(C) of the carbons C1 to C6, as seen in the following equation.
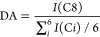
where C*i* represents the carbons C1 to C6. All DA values calculated are summarized in Table 1.

The signals from the lactic acid modification, C10 and C11, suffer from signal overlap by the chitin backbone and the chitin acetate group, respectively. To correct this, the integral of the sample without any modification, that is, ChNC_HCl, was used as a baseline. Using the same integral regions, 69.9–67.0 and 22.3–19.5 ppm, for both spectra and thus removing the integral of ChNC_HCl from the integral of the ChNC_lact, the corrected ChNC_lact integral *I*_corr_(C) was obtained. Dividing the corrected integrals with the average of all chitin signals provided the DS for the lactate modification as seen in the following equation
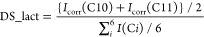


The butyrate signals C13 and C14, the latter partly overlapping with the acetate, could be used to obtain the integrals to calculate the DS_but. Even though the signals from the butyrate modification did not suffer from the same overlap issue as the lactate, also for this sample, the ChNC_HCl was used as a baseline. Analogous to above, the corrected ChNC_but integrals were obtained using the integral regions 39.3–35.5 and 21.8–18.0 ppm. The average of these integrals was then divided by the average of the integrals of the chitin backbone to obtain the DS for butyrate, as seen in the following equation.
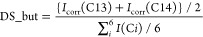


It should be noted that the C15 signal of the butyrate, even though being fully separated from other nearby signals, was not used to calculate the DS_but. This was due to the reduced relative integral of this freely moving methyl group with respect to the relaxation recovery delay.^63^

Using the average cross-section diameter of the ChNC rods for each material and assuming a circular cross-section of the rod with one chitin molecule layer on the surface, the SF could be calculated using the following equation
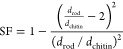
where *d*_rod_ is the average diameter of the chitin rod and *d*_chitin_ is the diameter of the chitin repeating unit, which has previously been found to be 0.688 nm.^64,65^ The SF values are summarized in Table 1. Dividing the obtained DS from the previous section by the recently calculated SF values provides DS_surf_, also summarized in Table 1. The DA and SF were determined with a precision of 5%.^51,53^

### Rheology

The rheological analysis was performed on an Anton Paar MCR702 TwinDrive rotational rheometer, using a transparent glass parallel plate geometry (diameter 43 mm) with a measurement gap of 0.6 mm. The tests were performed in a single motor–transducer configuration using the rheo-optical visualization setup of Fazilati *et al.*,^56^ based on the P-PTD200/GL accessory. Steady shear, dynamic strain sweep, and dynamic frequency sweep tests were performed at ambient temperature, 23 °C. The steady shear tests were performed at shear rates between 3 × 10^–3^ and 10^2^ s^–1^ using a custom procedure for steady-state detection. Dynamic strain sweep tests were performed at a constant angular frequency of 2 rad/s, and the strain amplitude was varied between 10^–2^ and 5 × 10^2^% to determine the limit of the linear viscoelastic regime. Thereafter, dynamic linear viscoelastic frequency sweeps were performed at constant strain amplitude for angular frequencies ranging between 8 × 10^–2^ and 2 × 10^2^ rad/s. The simultaneous PLI was performed perpendicularly to the shearing plane, with birefringence patterns observable through the transparent region of the upper geometry (approximately 7.5 mm from the geometry edge). The PLI setup was used in the transmission mode^39,56,57^ with the polarizer and analyzer being positioned at a 45° relative orientation (0, 45)°. PLI data were recorded in the form of HD format (1280 × 720 pi) video recordings (30 fps). More details about the custom setup can be found elsewhere.^54^ All tests were performed at room temperature (23 °C), and a relaxation time of 300 s was kept between setting the sample to the gap and starting a test. The suspensions at different concentrations were prepared by controlled dilution of the 10% hydrosols.

## Supporting Information Available

Schematic representation of chitin and cellulose chemical structures, scheme of the Fischer esterification mechanism, all the reaction conditions tested for chitin hydrolysis and one-pot acid hydrolysis/Fischer esterification, FT-IR spectra and XRD diffractograms of native chitin and of all the ChNCs obtained, deconvolution of XRD diffractograms for native chitin and butyrate-modified ChNCs, TEM, STEM, and AFM analysis of ChNCs used for the rheological study, steady shear viscosity functions and dynamic moduli from strain sweep for the samples ChNC_HCl and ChNC_HCl_s at different concentrations, birefringence patterns for the samples ChNC_HCl_s and ChNC_lact, and stability evolution over time of control emulsion oil-in-water (PDF)

## Abbreviations

ChNC: chitin nanocrystals
NMR: nuclear magnetic resonance spectroscopy
rheo-PLI: rheology coupled with polarized light imaging
CNC: cellulose nanocrystals
HCl: hydrochloric acid
p*K*_a_: acid dissociation constant
ChNC_HCl: chitin nanocrystals obtained through acid hydrolysis
AFM: atomic force microscopy
ChNC_but: butyrate-modified chitin nanocrystals
ChNC_lact: lactate-modified chitin nanocrystals
TEM: transmission electron microscopy
STEM: scanning electron microscopy on transmission mode
ATR FT-IR: attenuated total reflectance Fourier transform infrared
XRD: X-ray diffraction
DA: degree of acetylation
DS: degree of substitution
SS CP/MAS ^13^C NMR: solid-state cross-polarization magic angle spinning carbon-13 nuclear magnetic resonance
SF: surface fraction
CI: crystallinity index
BP: biphasic
LC: liquid crystalline
IG: isotropic gel
