# Supplementary material for: Green Topochemical Esterification Effects on the Supramolecular Structure of Chitin Nanocrystals: Implications for Highly Stable Pickering Emulsions
Source: ACS Appl Nano Mater. 2022 Apr 4;5(4):4731–43. doi: 10.1021/acsanm.1c03708 (PMC9039965; doi:10.1021/acsanm.1c03708)
Supplement: Supplementary file 1 — an1c03708_si_001.pdf [file an1c03708_si_001.pdf]

# Green Topochemical Esterification Effects on the Supramolecular Structure of Chitin Nanocrystals: Implications for Highly Stable Pickering Emulsions

*Chiara Magnani* [†,§], *Mina Fazilati* [∥,¶], *Roland Kádár* [∥,⊥], *Alexander Idström* [‡], *Lars Evenäs* [‡,⊥], *Jean-Marie Raquez* [†], *Giada Lo Re* [∥,⊥]*

† Laboratory of Polymeric and Composite Materials (LPCM), Center of Innovation and Research in Materials & Polymers (CIRMAP), University of Mons (UMONS), B-7000 Mons, Belgium

§ Laboratory of Proteomics and Microbiology, Research Institute for Biosciences, University of Mons (UMONS), B-7000 Mons, Belgium

∥ Department of Industrial and Materials Science IMS, Chalmers University of Technology, SE-412 96 Gothenburg, Sweden

⊥ Wallenberg Wood Science Center (WWSC), Chalmers University of Technology, SE-412 96 Gothenburg, Sweden

‡ Department of Chemistry and Chemical Engineering, Chalmers University of Technology, SE-412 96 Gothenburg, Sweden

**Scheme S1.** Chemical structure of cellulose and chitin

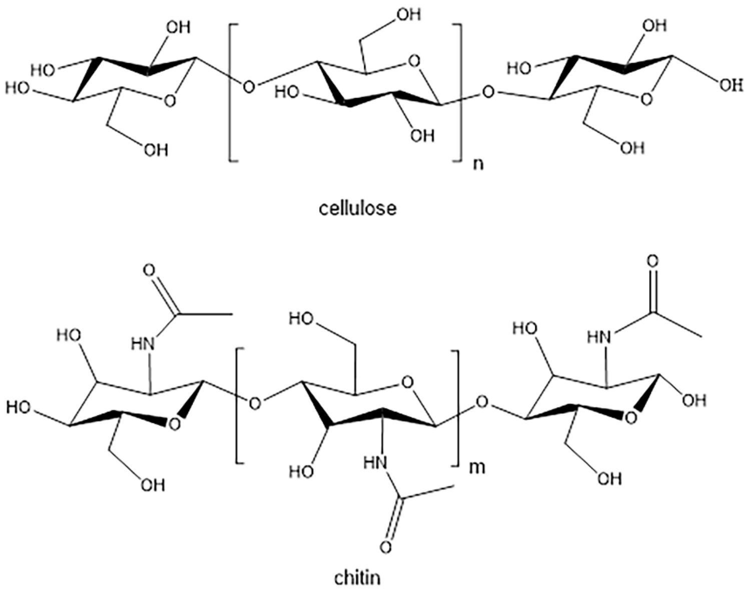

**Scheme S2**. Mechanism of Fischer esterification on chitin

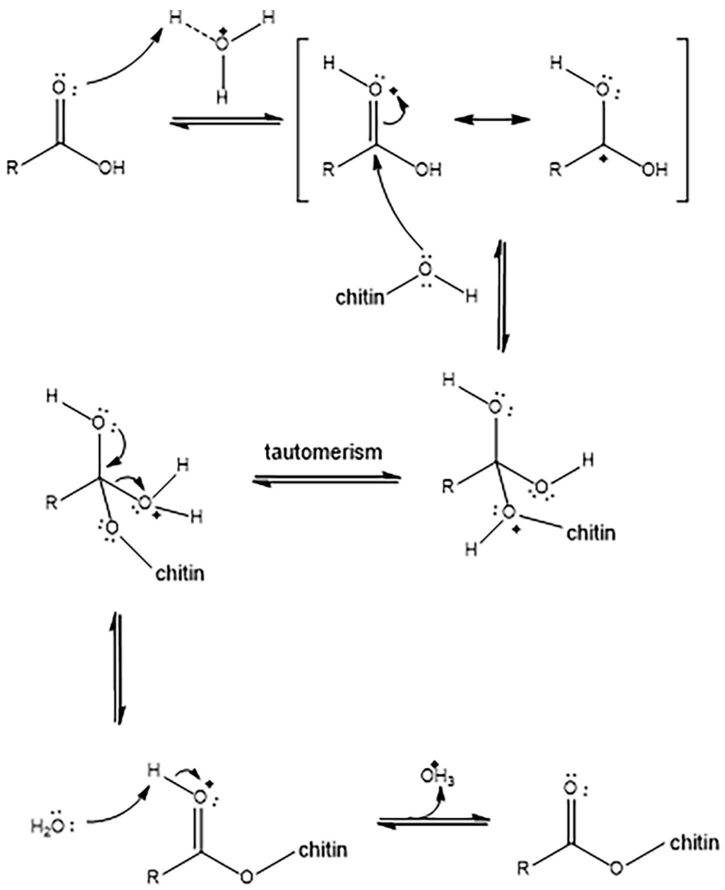

**Table S1.** List of chitin nanocrystals obtained at different synthesis conditions

| Acronym | code | HCl | time | temperature |
|---|---|---|---|---|
| **ChNC_HCl_s** | - | 3 M | 2 h | 105 °C |
| **ChNC_HCl** | - | 3 M | 1,5 h | |
| **ChNC_lact** | ChNC_lact_0.03-3 | 0,03 M | 3 h | Reflux (116 °C) |
| | ChNC_lact_0.03-5 | 0,03 M | 5 h | |
| | ChNC_lact_0.07-3 | 0,07 M | 3 h | |
| **ChNC_but** | ChNC_but_0.03-3 | 0,03 M | 3 h | Reflux (107 °C) |
| | ChNC_but_0.07-3 | 0,07 M | 3 h | |

Note: (ChNC_HCl_s: chitin nanocrystals obtained through HCl acid hydrolysis for 2 h; ChNC_HCl: chitin nanocrystals obtained through HCl acid hydrolysis for 1,5 h; ChNC_lact_0.07-3: chitin nanocrystals obtained in presence of lactic acid and HCl 0,07 M; ChNC_but_0.07-3: chitin nanocrystals obtained in presence of butyric acid and HCl 0,07 M; ChNC_but_0.03-3: chitin nanocrystals obtained in presence of butyric acid and HCl 0,03 M; ChNC_lact_0.03-3: chitin nanocrystals obtained in presence of lactic acid and HCl 0,03 M; ChNC_lact_0.03-5: chitin nanocrystals obtained in presence of lactic acid and HCl 0,03 M for 5 hours; where not specified the reaction has last 3 hours.).

The samples ChNC_lact_0.07-3 and ChNC_but_0.03-3 were selected for the topochemical and rheological characterization in comparison with ChNC_HCl and their acronyms in the main text were simplified in ChNC_lact and ChNC_but, respectively, for the sake of comprehension.

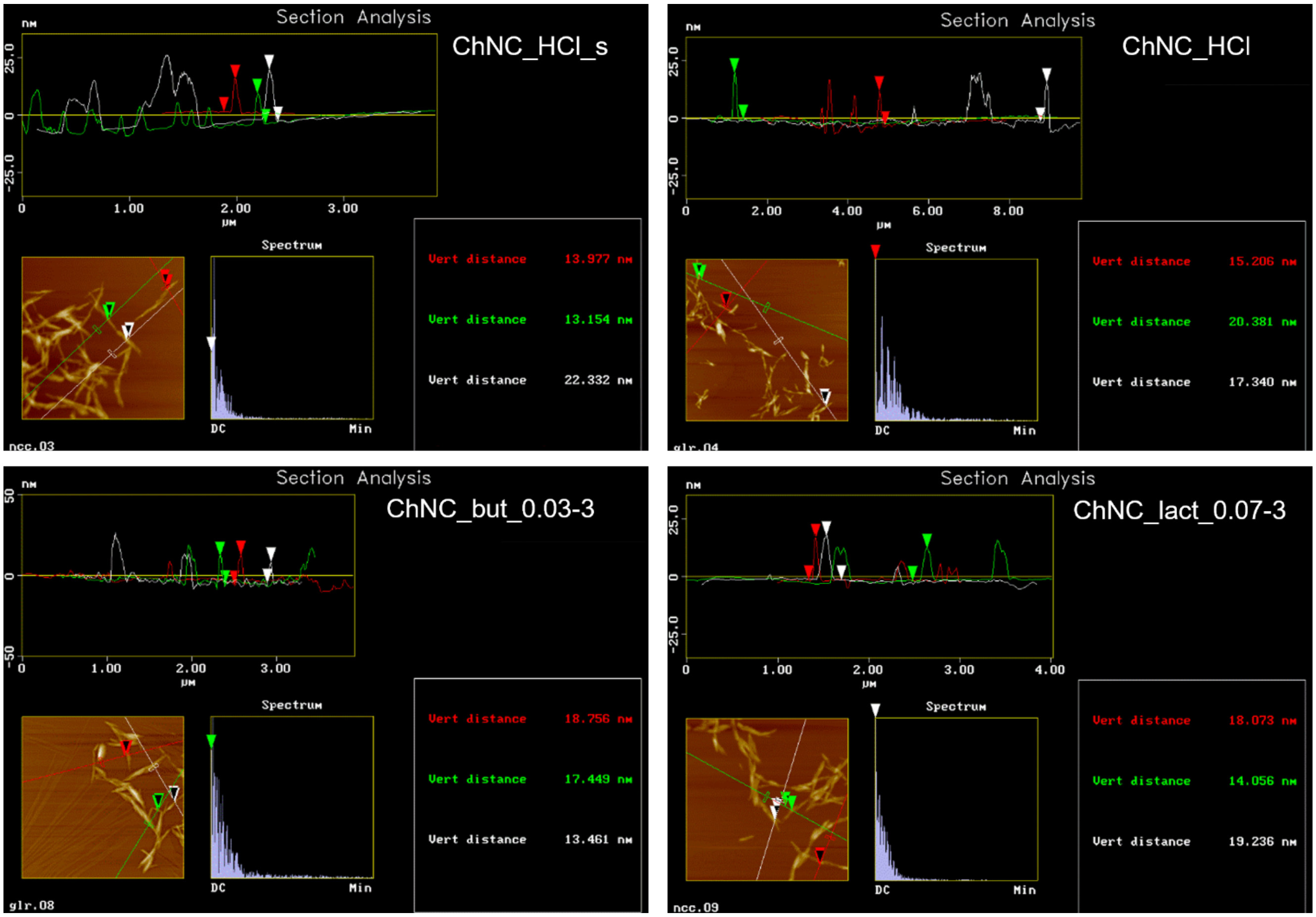


**Figure S1.** AFM analysis for the assessment of the diameter (indicated as vertical distance) of the different ChNCs selected for the study. In particular: ChNC_HCl_s and ChNC_HCl (top-leftand right images, respectively), and ChNC_but and ChNC_lat (bottom-left and right images, respectively).

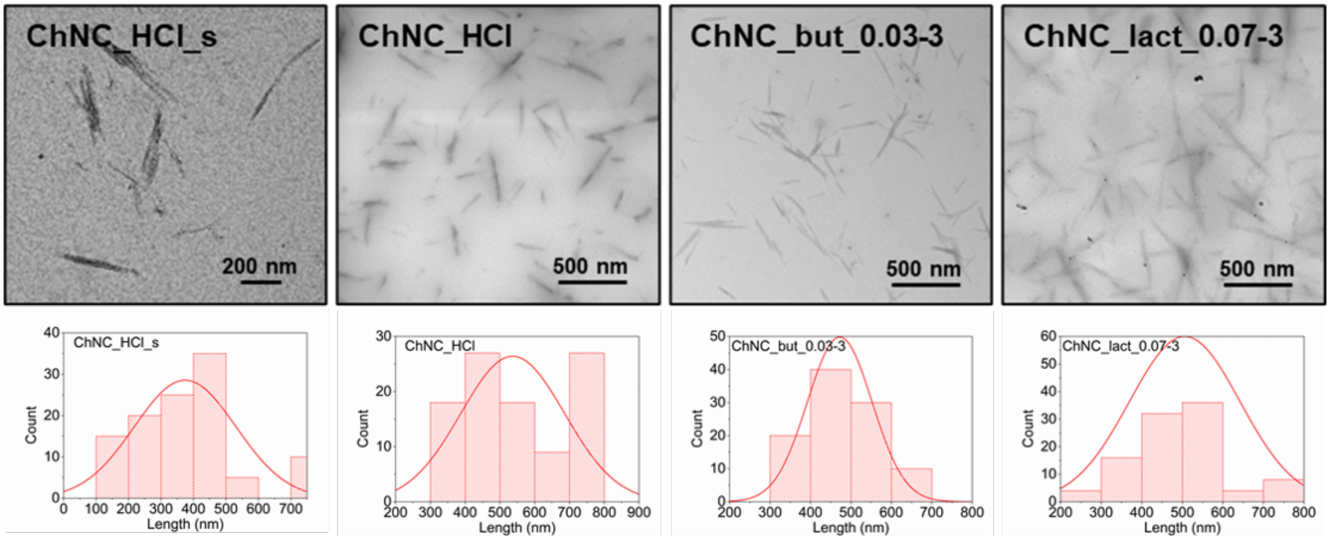


**Figure S2.** TEM (left top corner, bar 200 nm) and STEM (bars 500 nm) micrographs of chitin nanocrystals and related length distribution.

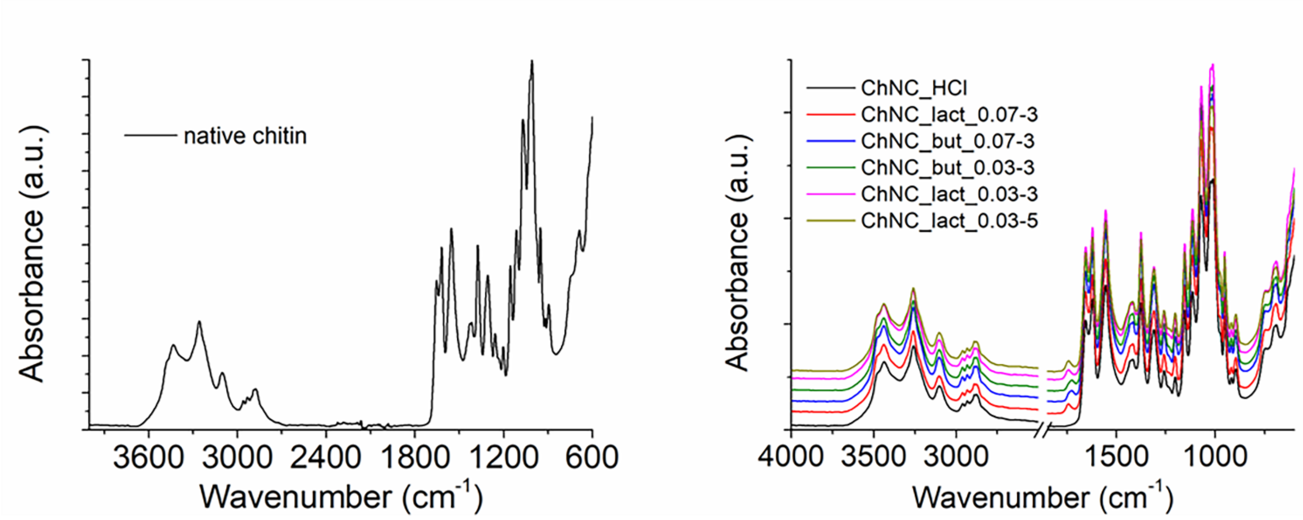


**Figure S3.** FT-IR spectra of native chitin and chitin nanocrystals

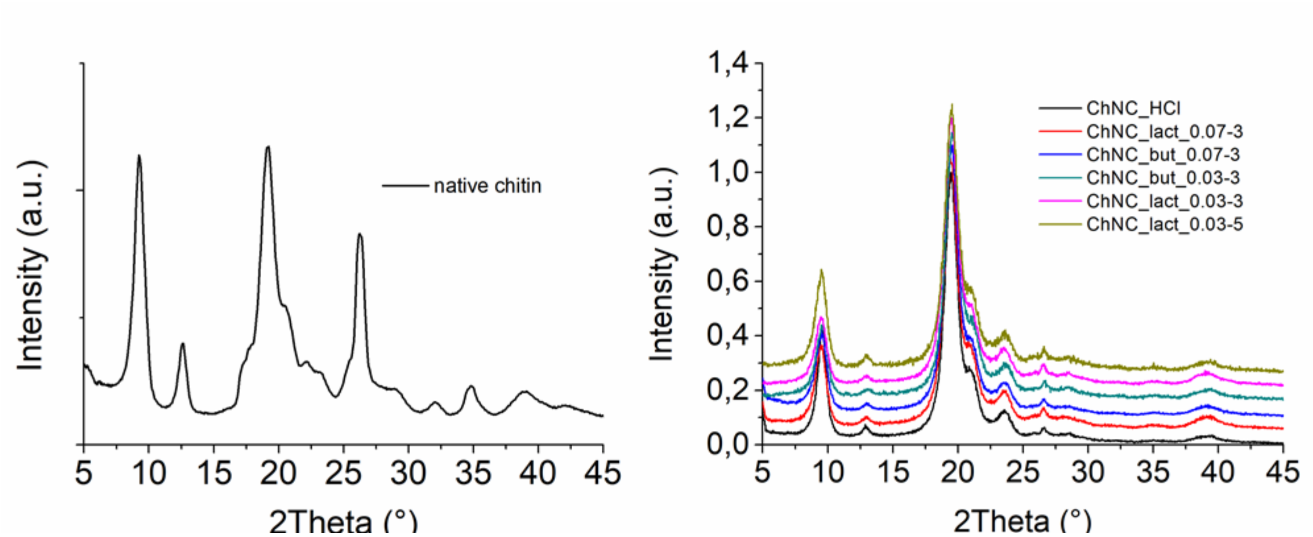


**Figure S4.** X Ray Diffraction analysis of native chitin and chitin nanocrystals

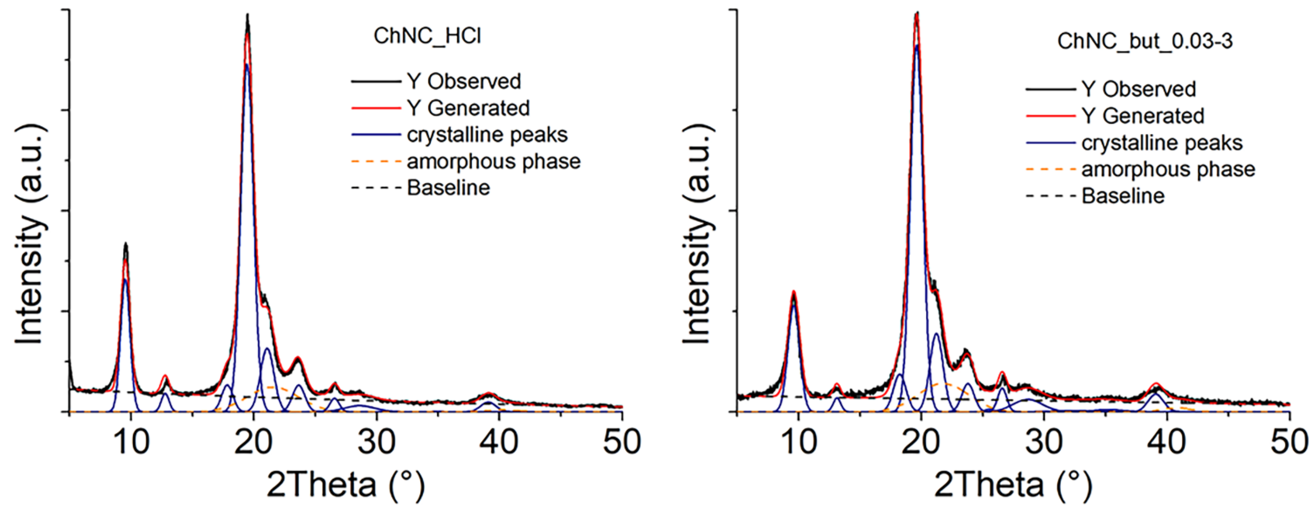


**Figure S5.** Deconvolution of DRX analysis using PeakFit program. Samples ChNC_HCl and ChNC_but_0.03-3 are shown as examples. For all deconvolutions the index $R^2$ was higher than 0,99.

**Table S2.** Summary of size and zeta potential (note to equilibrate the pH) for selected samples to investigate the influence of size and of chemical moieties.

| Acronym | L (nm) | D (nm) | L/D | zeta potential (mV) |
|---|---|---|---|---|
| **ChNC_HCl_s** | 374 ± 159 | 14 ± 2 | 27 | 41 ± 5 |
| **ChNC_HCl** | 537 ± 156 | 18 ± 3 | 30 | 30 ± 6 |
| **ChNC_lact_0.07-3** | 506 ± 138 | 17 ± 3 | 30 | 28 ± 6 |
| **ChNC_but_0.03-3** | 472 ± 84 | 17 ± 3 | 28 | 27 ± 4 |

## Rheology study: effect of ChNC dimensions

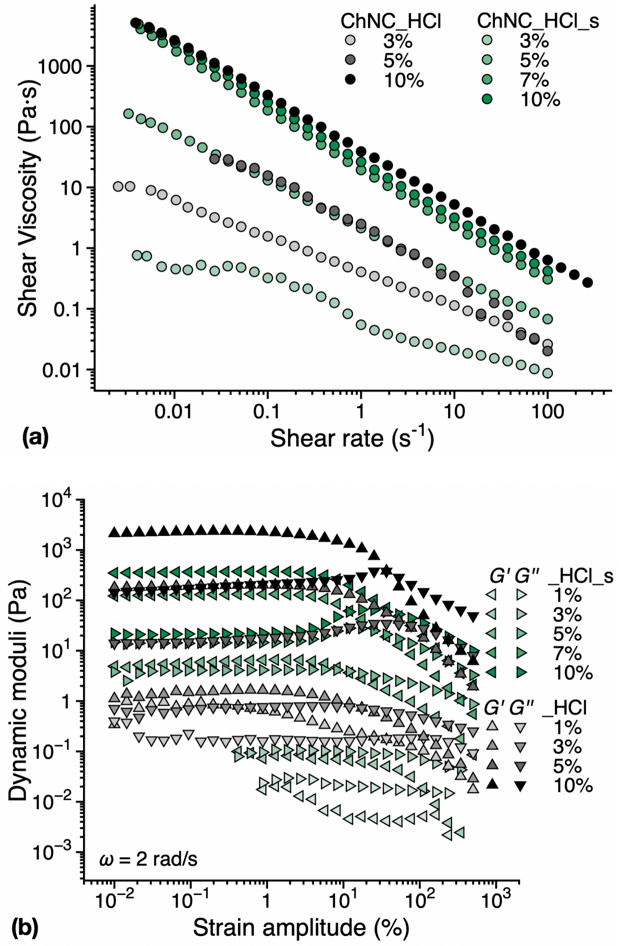


**Figure S6.** (a) Steady shear viscosity functions and (b) dynamic moduli from strain sweep tests showing the influence of ChNC_HCl size / aspect ratio, see Table S2, and concentration.

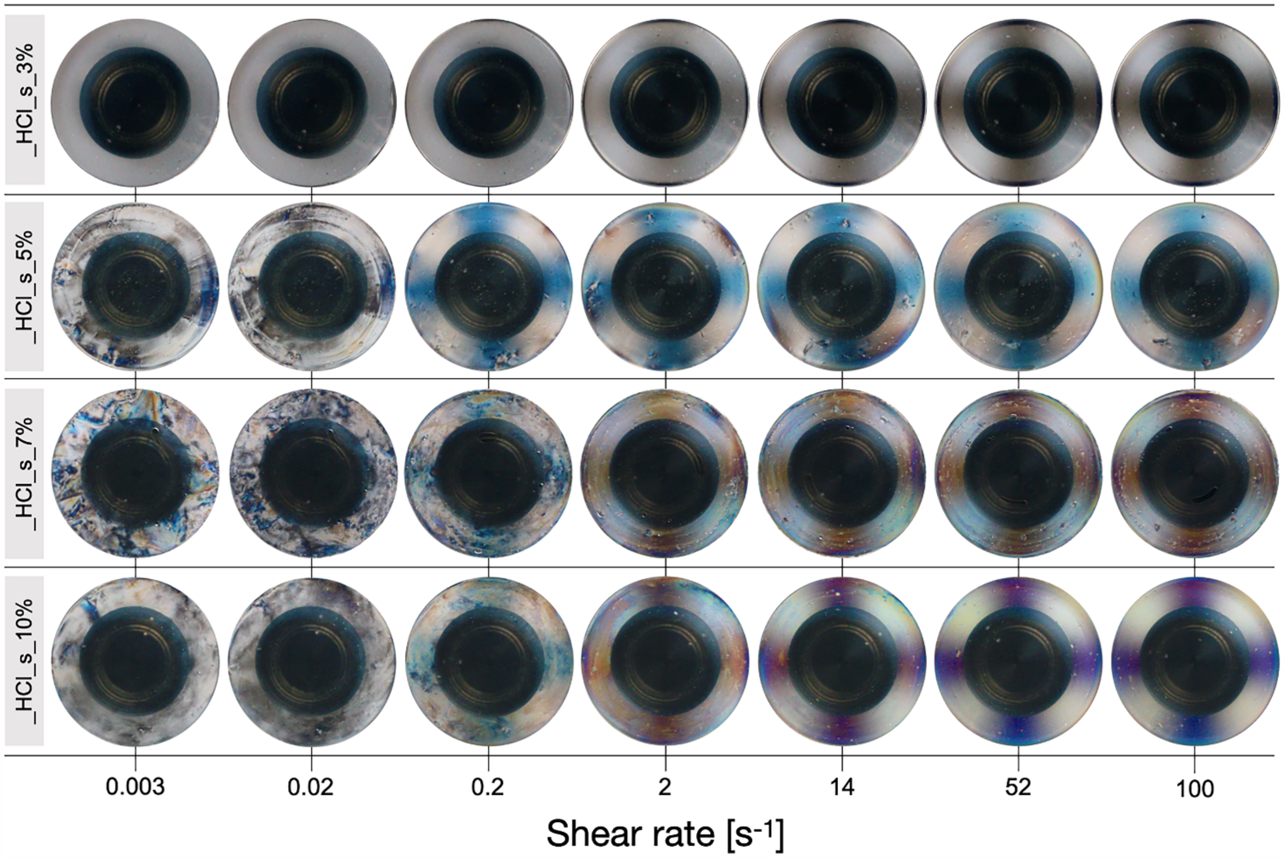


**Figure S7.** PLI still frames showing the circumferential birefringence patterns at selected shear rates from the steady shear tests showing the influence of concentration on ChNC_HCl_s.

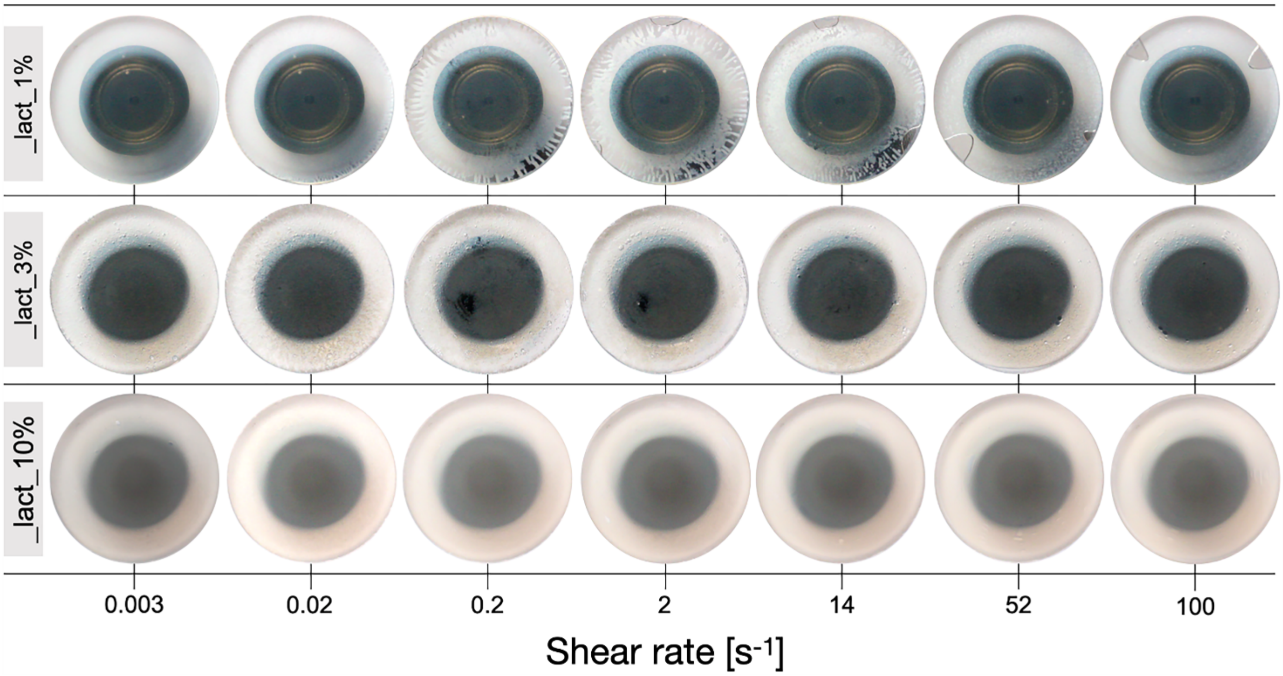


**Figure S8.** PLI still frames showing the circumferential birefringence patterns at selected shear rates from the steady shear tests showing the influence of concentration on ChNC_lact_0.07-3.

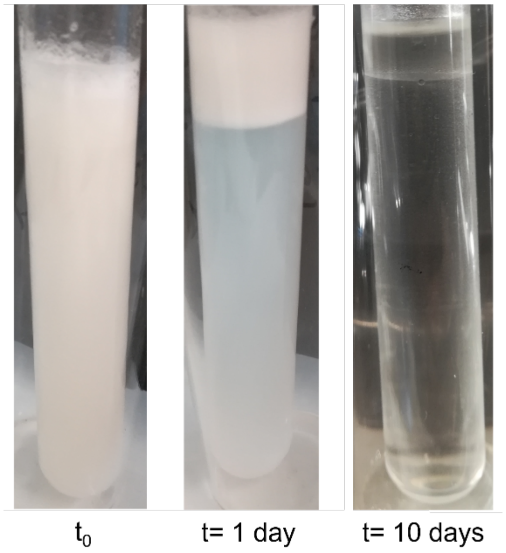


**Figure S9.** Control emulsion O/W after emulsification through tip sonication ($t_0$) and evolution after two minutes and one day.
